# Prevention of HPV-Related Oral Cancer by Dentists: Assessing the Opinion of Dutch Dental Students

**DOI:** 10.1007/s13187-017-1257-9

**Published:** 2017-07-24

**Authors:** Marcella R. Poelman, Henk S. Brand, Thymour Forouzanfar, Ellen M. Daley, Derk H. Jan Jager

**Affiliations:** 1 Centre for Special Care Dentistry (Stichting Bijzondere Tandheelkunde), Gustav Mahlerlaan 3004, Amsterdam, 1081 LA the Netherlands; 2 Department of Oral Biochemistry, Academic Centre for Dentistry Amsterdam (ACTA), Gustav Mahlerlaan 3004, Amsterdam, 1081 LA the Netherlands; 3 Department of Oral and Maxillofacial Surgery and Oral Pathology, Amsterdam Movement Sciences, VU University Medical Center, VU University, P.O. Box 7057, 1007 MB Amsterdam, The Netherlands; 4 Department of Community and Family Health, College of Public Health, University of South Florida, MDC 56, 13201 Bruce B. Downs Blvd, Tampa, 33612 FL USA; 5 Department of Oral Health Sciences - Prosthetics section, KU Leuven & University Hospitals Leuven, Box 7001, Kapucijnenvoer 7, Leuven, BE-3000 Belgium

**Keywords:** HPV, HPV vaccination, Head and neck cancer, Oral cancer, Prevention, Public health

## Abstract

The aim of this study is to assess dental students’ opinions of the dentists’ role in primary prevention of human papillomavirus (HPV)-related oral cancer using a cross-sectional web-based survey. A questionnaire, containing questions about knowledge of HPV and oral cancer, confidence in head and neck examination and role of the dentist in preventing HPV-related oral cancer, was sent to all students of the Academic Centre of Dentistry Amsterdam (*n* = 912). One hundred and twenty-six (*n* = 126) students completed the questionnaire. Significantly, more master students (75%) than bachelor students (54.3%) were aware that HPV is a causative factor for oral cancer. Master students had more knowledge of HPV than bachelor students, but knowledge about HPV vaccination was irrespective of the study phase. The majority of dental students agreed that it is important to discuss HPV vaccination with patients. Eighty-nine percent of the students think that more education about symptoms of oral cancer will increase screening for oral cancer. Development of a protocol for screening in dental practices was considered even more important. According to dental students, dentists should discuss HPV as a risk factor for oral cancer with patients. Future dentists are willing to be involved in both primary and secondary prevention of HPV-related oral cancer. Therefore, screening for oral cancer and education about HPV vaccination should be integral elements of the dental curriculum.

## Introduction

Head and neck cancer is the sixth most common cancer worldwide, with an annual incidence of approximately 600,000 cases [1, 2]. The most common cancer in the head and neck area is the squamous cell carcinoma. In the past, head and neck cancer was most commonly seen in older adults with a history of tobacco and alcohol use. Due to a decrease in tobacco use, the number of newly diagnosed tobacco-related head and neck cancers is declining. However, the overall number of patients with head and neck cancer is still increasing, especially of patients with squamous cell carcinomas of the oropharynx [3]. Nowadays, patients diagnosed with head and neck cancer are more likely to be younger middle-aged men who may lack the previously significant risk factors as tobacco and alcohol use. These changes are related to the human papillomavirus (HPV) [4]. The prevalence rates of HPV-positive oropharyngeal cancers have increased significantly over the last decades. HPV is the most common sexually transmitted virus, so one of the explanations of the increased prevalence rates may be a change in sexual behaviour [5]. Engaging in orogenital sex with multiple sex partners is associated with HPV-related oral cancer [6].

The prevalence of HPV-related oropharyngeal cancer varies from 20 to 90%. The highest rates are reported in North America and Asia; the reported prevalence in Europe is usually lower [7–9]. This variation may be related to lack of a standardized HPV detection method, varying exposures to HPV in different geographical regions and referral bias in the populations tested [5, 10].

There are many different sub-types of the HPV virus. The majority of HPV infections are asymptomatic and resolve spontaneously within 2 years. Persistent infection with a ‘high-risk’ sub-type is a risk factor for the development of cancer in various regions such as the oropharynx, cervix, anus, and penis. Regardless of anatomic site, most of these cancers are associated with HPV types 16 and 18 [11].

HPV-positive carcinomas are considered to be a different tumour entity, based on prominent biological and etiological differences, when compared with HPV-negative carcinomas [12]. In addition, HPV-positive carcinomas have a better response to therapy, lower rates of adverse events, and better overall survival [13].

The World Health Organization (WHO) recommends HPV vaccination to be included in national immunisation programmes [14] with the specific aim of protecting women against cervical cancer. The use of HPV vaccines is not recommended yet to prevent HPV-positive head and neck carcinomas. The two currently available HPV vaccines prevent transmission of HPV types 16 and 18, the two strains attributable to 90–95% of HPV-positive oropharyngeal carcinomas. So hypothetically, the use of these HPV vaccines may cause a reduction in the increasing incidence of oropharyngeal cancer [15, 16].

In the Netherlands, the HPV vaccine Cervarix is offered free of charge to preadolescent girls, and uptake has been fairly consistent between 50 and 60% over the past 5 years. When girls are vaccinated, heterosexual men could benefit indirectly from a reduced transmission of vaccine type HPV [17]. However, including boys in the HPV vaccination programme might be a more cost-effective strategy for the prevention of HPV-related cancer (oropharyngeal and anal) in the general population [17, 18].

A systematic review of girls’ and parents’ information needs and views has shown that knowledge about the vaccination is poor, and there are many misconceptions [19]. The association between individual knowledge and HPV vaccination makes providing information essential to increase uptake [20]. Therefore, health care providers must be prepared to provide patients with information on HPV vaccination and discuss the sexual transmission of HPV [21, 22]. In Florida, dentists are willing to play a role in primary prevention of HPV-related oral cancer, despite lack of high levels of knowledge [23, 24].

Dentists are among the most visited health care providers. In the Netherlands, almost 80% of the population visits the dentist annually. Clinical screening for oral cancer is a form of secondary prevention and is an important part of dental examination, because early diagnosis of (pre)malignant lesions increases the probability of cure [25]. Screening has been shown to be an effective and cost-effective way of improving early detection [26]. However, over 60% of oral cancers are diagnosed late, and many medical students report a lack of confidence in screening head and neck for cancer [27].

As the number of patients treated for HPV-related oral cancer increases, it is likely that dentists will be asked questions that were previously considered taboo and potentially cause embarrassment. The possible psychosocial impact of diagnosis of HPV-related oral cancer should not be overlooked either. So dentists need to develop advanced communication skills to address these topics [28].

It could be difficult to achieve a preventative role in HPV infection for dentists, because it requires public recognition and professional acceptance. Professional organisations can enable advancement by providing the profession with information and tools. Strengthening content on this topic in the dental curriculum may also be beneficial [23, 29, 30].

In contrast to other European countries and the USA, dental professional organisations in the Netherlands do not yet support the dentists’ role in prevention of HPV-related oral cancer [31]. Furthermore, it is not yet known what role dentists in the Netherlands see for themselves. Therefore, the aims of this study among dental students were (1) to assess awareness of the association between HPV and oral cancer, (2) to explore their readiness for playing a role in primary prevention of HPV-related oral cancer by discussing the HPV vaccine with patients, and (3) to assess their confidence in screening the oral cavity for (pre)malignant lesions.

## Methods

### Study Design

A cross-sectional web-based research was performed, using a 19-item questionnaire, based on a validated questionnaire from a previous study among dentists in the USA [23]. This questionnaire was translated, reformulated according to regulations for dentists in the Netherlands, and adapted for dental students. Dental education, in the Netherlands, comprises a 3-year bachelor programme and a subsequent 3-year master programme. Both programmes have to be completed before one can register as a dentist according to the Professionals in Individual Health Care Act of the Netherlands. Next to the master in dentistry degree (MSc), there are two dental-specialists recognized by the Ministry of Education, Culture and Science of the Netherlands: maxillofacial surgery and orthodontics. Furthermore, there are a number of board certified postgraduate programmes such as (maxillofacial) prosthodontics, periodontics, pedodontics, gnathology, endodontics, and special care dentistry. The postgraduate programmes are recognized by the related scientific associations but not recognized as dental specialists. A preliminary version of the questionnaire was tested on two dental students. Their feedback led to some small adjustments of the questionnaire. The results of these two students were not included in the statistical analysis. The final version of the questionnaire took approximately 5–10 min to complete.

#### Instrument

To assess students’ knowledge of HPV and the HPV vaccine, statements used required one of the following responses: ‘correct’, ‘incorrect’, or ‘I do not know’. Items included were (1) awareness of relation between HPV and oral cancer, (2) risk factors for oral cancer, (3) transmission of HPV, (4) target group for vaccination, and (5) safety of the HPV vaccine.

Furthermore, 4- and 5-point Likert scales were used to assess students’ opinion about (1) their skills for screening for oral cancer, (2) need to develop professional guidelines, (3) discomfort discussing sexual history topics with patients, and (4) the role for dentists regarding primary prevention of HPV-related oral cancer. All items translated from the previous questionnaire [22] were maintained on the original 4-point Likert scales to enable comparison. Items added to the original questionnaire were on five-point Likert scales, as these have a higher reliability than the four-point version [32, 33].

Multiple-choice questions, with the possibility of selecting multiple options, contained items about current education about HPV and factors motivating dentists to discuss HPV vaccination with patients. Additionally, demographic variables (e.g., sex, ethnicity, age, year in dental school, received HPV vaccine) were included.

#### Data Collection and Analyses

The questionnaire was administered via Cognito Forms (Cognito, Columbia, SC, USA), using a universally accessible web address, and an electronic invitation was emailed to all registered students of the Academic Centre of Dentistry Amsterdam (*n* = 912). Student participation was voluntary, and responses were processed anonymously. After 3 and 4 weeks, students received an email with a reminder requesting participation in the survey.

Data were analysed with SPSS, version 22.0 (IBM Corp, Armonk, NY, USA) using unpaired *t* tests and Chi-square goodness of fit test to investigate the difference in responses between bachelor and master students. When the requirement of a minimum of 5 or more expected frequencies in each category was not met, a Fisher’s exact test (FET) was used. Differences in responses between subgroups, on questions with categorical data, were assessed using the Mann–Whitney *U* test. A *p* value of 0.05 or lower was considered statistically significant.

## Results

The total number of registered dental students at October 1st 2015 was 914. The survey was emailed to their student-email account, and two emails were returned ‘undeliverable’. A response of 126 surveys was obtained, resulting in a response rate of 14%. Respondents were primarily female (68.3%), and one third of the female students were vaccinated against HPV (31.7%). Approximately half of the respondent students were in the bachelor programme (first 3 years of a 6-year curriculum) (55.6%).

**Fig. 1:**
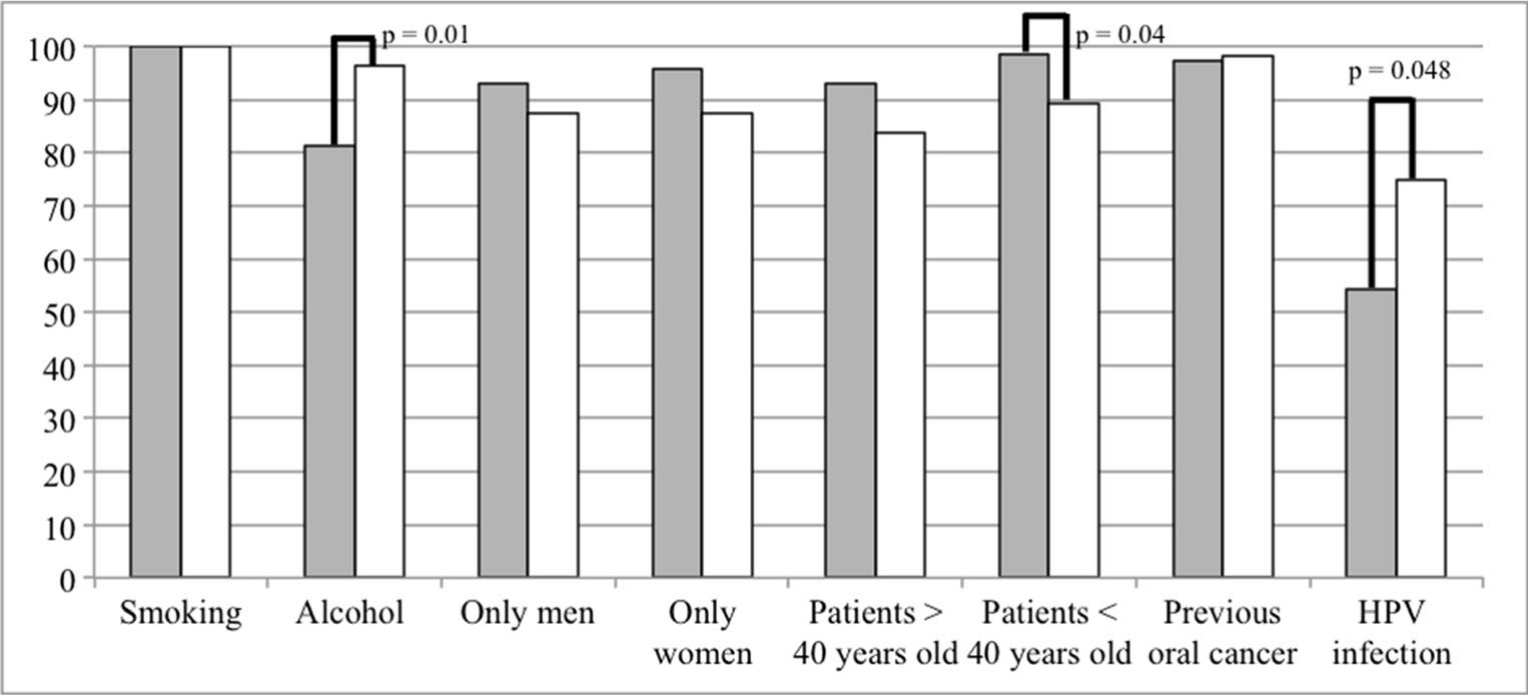
Percentages correct answers to questions on potential factors of oral cancer. Significant differences between Bachelor and Master students are indicated

Before participation in the present survey, 63.5% of the students were aware of the relation of HPV with oral cancer. Significantly, more master students (75.0%) than bachelor students (54.3%) had this knowledge (*χ* ^2^ (2, *N* = 126) = 6.08, *p* = 0.048) (Fig. 1). Seventy percent of the bachelor and 75% of the master students reported knowledge about availability of HPV vaccination. Female students (75.6%) were more often aware of the fact that in the Netherlands, HPV vaccine is only available for girls, compared to men (52.5%) (*χ* ^2^ (2, *N* = 126) = 12.7, *p* = 0.002). More female students who had been vaccinated against HPV answered more knowledge questions correctly than unvaccinated female students, but this was not significant (*χ* ^2^ (2, *N* = 126) = 0.40, *p* = 0.82).

Of the 16 questions assessing dental students’ knowledge about HPV, five questions (# 1, 4, 5, 7, 9) were answered incorrectly by more than 50% of the students. Master students knew significantly more often the correct answer on six items than bachelor students (*p* < 0.05; # 3, 4, 6, 8, 11, 13) (Table 1).

**Table 1:** Sixteen questions assessing dental students’ knowledge about HPV stratified for bachelor and master students. The correct answers for each item are indicated with an asterisk. Data are expressed as percentages

*N* = 126	Bachelor *n* = 70 (%)			Master *n* = 56 (%)			*χ* ^2^ (1, *N* = 126)
	Correct	Incorrect	I do not know	Correct	Incorrect	I do not know	
1. Approximately 50% of patients who get oral cancer will die from this disease	22.9	41.4*	35.7	26.8	46.4*	26.8	1.16, *p* = 0.561
2. Some types of HPV cause oral cancer	84.3*	1.4	14.3	89.3*	1.8	8.9	0.87, *p* = 0.649
3. Oral cancer is often preceded by the presence of clinically identifiable premalignant changes	70*	5.7	24.3	94.6*	3.6	1.8	13.67, *p* = 0.01
4. An increasing number of patients diagnosed with oral cancer lack risk factors as tobacco and alcohol use	28.6*	18.6	52.9	41.1*	35.7	23.2	11.80, *p* = 0.03
5. The average age of patients diagnosed with oral cancer is declining	38.6*	10	51.4	46.4*	21.4	32.1	5.85, *p* = 0.054
6. The majority of malignant lesions in the oral cavity is diagnosed in an advanced stage of progression	54.3*	7.1	38.6	83.9*	8.9	7.1	16.67, *p* < 0.001
7. There are more than 100 types of HPV	30*	12.9	57.1	35.7*	19.6	44.6	2.16, *p* = 0.34
8. A person can have HPV without knowing it	81.4*	0	18.6	96.4*	0	3.6	6.68, *p* = 0.012
9. Most HPV infections resolve within a short time	17.1*	37.1	45.7	16.1*	53.6	30.4	3.80, *p* = 0.15
10. Some types of HPV cause cervical cancer	74.3*	4.3	21.4	85.7*	3.6	10.7	2.70, *p* = 0.26
11. HPV causes herpes and cold sore	27.1	47.1*	25.7	32.1	60.7*	7.1	7.49, *p* = 0.024
12. HPV causes HIV/aids	2.9	75.7*	21.4	3.6	89.3*	7.1	4.96, *p* = 0.084
13. HPV is a sexually transmitted virus	67.1*	11.4	21.4	83.9*	10.7	5.4	6.18, *p* = 0.033
14. Antibiotics can cure a HPV infection	10	61.4*	28.6	8.9	73.2*	17.9	2.19, *p* = 0.34
15. There is a vaccine that prevents against certain types of HPV	65.7*	10	24.3	78.6*	12.5	8.9	5.10, *p* = 0.078
16. Using a condom decreases the chance of transmitting HPV	61.4*	10	28.6	76.8*	7.1	16.1	3.48, *p* = 0.176

Of the six questions testing the knowledge about the HPV vaccine (Table 2), two items (#5 and 6) were answered correctly by only one third of bachelor and master students (38.6, 31.4, 30.4, and 30.4% respectively).

**Table 2:** Six questions assessing dental students’ knowledge about HPV vaccination stratified for bachelor and master students. The correct answers for each item are indicated with an asterisk. Data are expressed as percentages

*N* = 126	Bachelor *n* = 70 (%)			Master *n* = 56 (%)			*χ* ^2^ (1, *N* = 126)
	Correct	Incorrect	I do not know	Correct	Incorrect	I do not know	
1 The vaccine prevents transmission of some types of HPV	51.4*	24.3	24.3	55.4*	25	19.6	0.40, *p* = 0.819
2. The HPV vaccine protects women against cervical cancer	71.4*	10	18.6	67.9*	16.1	16.1	1.07, *p* = 0.585
3. Individuals vaccinated against HPV do not have to practice safe sex (e.g., using condoms)	0	90*	10	0	92.3*	7.1	0.32, *p* = 0.572
4. In the national immunization programme the HPV vaccine is only available for females	64.3*	10	25.7	73.2*	8.9	17.9	1.27, *p* = 0.527
5. Men can request their general practitioner for HPV-vaccination; however, this is not covered financially	38.6*	1.4	60	30.4*	10.7	58.9	5.44, *p* = 0.066
6. The HPV vaccine is only effective for individuals who have never had sex before	37.1	31.4*	31.4	39.3	30.4*	30.4	0.061, *p* = 0.970

A large majority of the students were aware of the risk factors for oral cancer; all students correctly identified tobacco, 81% of the bachelor and 96% of master students identified alcohol consumption, and almost all students (bachelor students 97%, master students 98%) identified previous oral cancer as risk factors (Fig. 1). Significantly, more master students acquired their knowledge from theoretical education at the dental school (80.4%) compared to bachelor students (47.1%) (*χ* ^2^ (1, *N* = 126) = 14.6, *p* < 0.001). Internet and social media and professional literature are relatively important resources for acquiring information for dental students (33.3 and 34.1%, respectively). Clinical practice was hardly a source of information for students (4%).

Dental students reported a low level of confidence in performing a screening for oral cancer. On a 4-point Likert scale (1 = very confident, 4 = not confident), students rated their confidence in visual inspection on average at 3.2 (SD = 0.8) and manual palpation on average at 3.5 (SD = 0.6).

To stimulate students to perform a screening for oral cancer in all patients, a large proportion of the students would like to have additional training during their education (88.9%). Furthermore, availability of reliable screening devices was suggested (50.8%) as well as enhancement of knowledge about HPV and oral cancer in the general public (42.9%). According to students, the best way to inform patients about HPV was to present it as a risk factor for oral cancer (65.1%) followed by presenting HPV as an infectious disease (20.4%). Incorporation of a question into the written medical protocol about the sexual history of the patient was only recommended by 14.3% of the students. Discussing personal topics with the patient, such as lifestyle and substance abuse, were considered ‘easy’ (*mean* = 3.9, SD = 0.9) and *mean* = 3.6 (SD 0 = 0.9) respectively on a 5-point Likert scale from 1 = not easy to 5 = very easy. Topics as domestic violence, sexually transmitted infections, and eating disorders were considered ‘less easy’ to talk about (respectively *mean* = 2.2 (SD = 0.8); *mean* = 2.5 (SD = 0.9); *mean =* 2.6 (SD = 1.0)). Female students experienced significantly more discomfort in discussing these topics than male students (*p* < 0.05), except for domestic violence (Table 3). One hundred eleven students in our cohort were born in the Netherlands and 15 not (all non-Western; i.e., born outside the European Union). From 40 students, one or both parents were non-Western. Non-Western students or students with non-Western parents did not experience more discomfort in discussing these issues than Dutch students (Table 3).

**Table 3:** Differences between responses to five questions assessing how easy it is to discuss personal topics with the patient reported on a 5-point Likert scale (from 1 = not easy to 5 = very easy) by male and female students, by students born in the Netherlands and non-Western students and by students with parents born in the Netherlands and students with non-Western parents. For every sub-group of participants, the mean score ± SD and *p* values are presented

Question	Men	Women	*p*	Born in the Netherlands	Non-Western	*p*	Parents born in the Netherlands	Non-Western parents	*p*
1. Lifestyle	4.2 (±1.0)	3.7 (±0.9)	0.005	3.9 (±0.9)	3.6 (±1.1)	0.25	3.9 (±0.9)	3.8 (±1.0)	0.42
2. Domestic violence	2.3 (±0.8)	2.1 (±0.7)	0.08	2.1 (±0.7)	2.3 (±1.0)	0.64	2.1 (±0.8)	2.2 (±0.8)	0.58
3. Eating disorders	2.9 (±1.0)	2.5 (±0.9)	0.03	2.6 (±0.9)	2.9 (±1.3)	0.32	2.6 (±0.9)	2.7 (±1.1)	0.63
4. Sexually transmitted infections	2.8 (±0.9)	2.4 (±1.0)	0.02	2.5 (±0.9)	2.6 (±1.2)	0.69	2.5 (±0.9)	2.5 (±1.0)	0.76
5. Substance abuse	3.9 (±0.7)	3.5 (±1.0)	0.04	3.6 (±0.9)	3.7 (±1.2)	0.42	3.6 (±0.9)	3.6 (±1.1)	0.94

According to dental students, it is important for dentists to discuss HPV as a risk factor for oral cancer on a 5-point Likert scale of 1 = very important to 5 = not important (*mean* = 2.1, SD = 0.8). A significant difference was found in responses to this question between students who were born in the Netherlands (*mean =* 2.2, SD *=* 0.8) and non-Western students (*mean =* 1.6, SD = 0.6) (*U* = 565, *p* = 0.03, *r* = −0.2). Also, a significant difference was found when the students were divided based on whether their parents were born in the Netherlands (*N* = 86; *mean* = 2.2, SD = 0.8) or non-Western (*N* = 40; *mean* = 1.7, SD = 0.7) (*U* = 1275, *p* = 0.009, *r* = −0.2). These results suggest that non-Western students or students with non-Western parents, in our sample, are equal or more willing to discuss HPV with patients compared to Dutch students.

Dental students considered a protocol for oral cancer screening very important (*mean* = 1.68, SD = 0.63) on a 5-point Likert scale of 1 = very important to 5 = not important. The development of a protocol was considered significantly more important for female (*mean* = 1.55, SD = 0.61) than for male (*mean* = 1.95, SD = 0.60*)* students (*U* = 1138, *p* = 0.01, *r* = −0.3).

## Discussion

The prevalence rates of HPV-positive oral cancers are increasing rapidly, and the demographic profile of patients with oral cancer is changing [5]. The dentists may be a key health care provider for prevention of HPV-related oral cancer in patients. Discussing HPV as a risk factor, providing information about sexual behaviour to prevent infection and early detection of (pre)malignant lesions, might help to stop the increase in prevalence of HPV-positive oral cancers.

As dental students are future dentists, their opinion about the dentists’ role in primary prevention of oral cancer is important. To fulfil this role in the future, adequate education of dental students is essential. Several studies have demonstrated that medical students have insufficient knowledge of oral cancer. Recent studies in America showed poor baseline knowledge among medical students, with only 18 to 59% and 44 to 67% correctly identifying alcohol consumption and tobacco as risk factors for oral cancer. Less than a quarter (24%) of the medical students correctly identified HPV as a potential risk factor [27, 34]. The results from the Dutch dental students did not corroborate this knowledge deficit: 100, 88, and 64% named tobacco, alcohol, and HPV as risk factors for oral cancer. Although the dental students’ knowledge about HPV as risk factor for oral cancer was reasonable, basic knowledge about HPV was considerably less. One third of the knowledge questions of HPV were answered correctly by less than 50% of the students (Table 1). They did not know that the average age of patients diagnosed with oral cancer is declining, and patients are more likely to lack risk factors such as alcohol and tobacco use. This information is crucial in diagnosis of oral lesions. Master students answered significantly more items correctly than bachelor students. This may be related to the incorporation of theoretical education about HPV in the master curriculum. This suggestion is supported by the fact that 80% of the master students reported that the theoretical education of their dental school is their source of information about HPV. These findings suggest that theoretical knowledge about HPV of future dentists is reasonable.

Students who are vaccinated against HPV did not have more knowledge about HPV than non-vaccinated students. In the Netherlands, girls are invited for HPV vaccination at an age of 13 years (www.rijksvaccinatieprogramma.nl) and therefore need approval of their parents. Maybe the opinion and knowledge of their parents about vaccination plays a more important role in the decision to be vaccinated than their own opinion. It is also possible that both parents and girls have limited understanding about the HPV vaccine, when they decide whether or not to take the vaccination [19].

In America, 66% of medical schools do not include screening for head and neck cancer in their curricula. Even when students learn to perform this screening, the quality of this teaching is inconsistent [27]. It is likely that this contributes to the fact that 47% of medical students in America reported feeling ‘not very confident’ or less in examining the oral cavity for oral cancer [27]. The Dutch dental students reported the same lack of confidence. Insufficient education at dental schools may explain the reported lack of screening skills for oral cancer in dentists [29]. It also explains why a large majority of dental students (89%) suggested inclusion of more clinical training in screening for oral cancer in their curriculum. Education which contains discussions on HPV and clinical training by experienced dentists or oral surgeons has shown to be effective [27]. These educational methods have also been successful in educating dental students on the human immunodeficiency virus (HIV) [35].

Dental students express a need for development of protocols for screening for oral cancer. This opinion is shared by dentists in Florida [23]. Dental professional organisations could help in the development and introduction of these protocols. Patient information leaflets relating to the topic may also be a tool for providing accurate information. Furthermore, investment in advanced communication skills courses for dentists will help the practitioner in addressing sexual-related topics that were previously considered a taboo.

Although dentists in Florida stated that their profession had a clear role and responsibility in discussing the relation between oral cancer and HPV with patients, they were not ‘ready’ to discuss the HPV vaccine with their patients [23, 29]. On the contrary, Dutch dental students thought dentists should discuss this subject with their patients, which suggests students are ready to discuss the HPV vaccine with their patients. A possible explanation for this result is the difference in age of the subjects. Also cultural differences between the Netherlands and the USA may contribute to the difference in results. Furthermore, the American data were obtained in 2011 and 2013. Recent epidemiological findings about HPV and oral cancer may have contributed to dental students’ willingness in discussing HPV with patients. For example, the British Dental Association launched a campaign in April 2015 to increase knowledge of the relation between HPV and supported gender-neutral HPV vaccination.

A similar change in readiness of dentists to inform patients about tobacco and alcohol as risk factors for oral cancer has also been reported [36].

Dentists in America reported liability concerns and discomfort in having sexual health-related discussions with patients as a barrier for discussing HPV with patients [23, 29].

Research has shown that cultural background and religion could influence discomfort in having sexual health-related discussions [37, 38]. However, our study did not find differences in discussing this topic between students with a western and a non-Western ethnicity. Non-western students and students with non-Western parents were even more willing to discuss HPV with patients.

The study design in the previous study among dentists in Florida was guided by the transtheoretical model, to construct the stages of change to assess behavioural readiness of dentists to discuss HPV with patients for primary prevention of cancers [23]. As dental students are not yet treating patients independently, the outcome variable of this study was to assess the opinion of dental students about discussing HPV with patients. Therefore, it was not possible to segment them into stages of behavioural adaptation.

Another limitation of the present study is the potential risk of selection bias; since the participation was on a voluntary base, the study may have attracted students with a baseline level of knowledge that differs from that of their non-participating peers. However, the participation of female students in this study of 68% resembles the participation of female students at the Academic Centre for Dentistry Amsterdam of approximately 65%. Moreover, the relatively low response rate limits the generalizability of study results, although the response rate (14%) was higher than the response of dentists in Florida in a similar study (8%). Finally, as there are three dental schools in the Netherlands, these results only resemble the students’ opinions in Amsterdam, which may differ from their peers at universities in other parts of the country.

## Conclusion

Findings from the present study highlight that future dentists are willing to play a role in preventing HPV-related oral cancer. They might play this preventative role by (1) informing patients about HPV to reduce the risk of getting infected, (2) discussing HPV-vaccination, and (3) early detection of (pre)malignant lesions, which improves the prognosis of patients with oral cancer. To prepare dental students for this future professional role, dental schools should include more training on this topic in their curricula.
